# Association between platelet count and 30-day mortality in community-acquired pneumonia patients receiving systemic glucocorticoids therapy

**DOI:** 10.1038/s41598-026-46565-z

**Published:** 2026-04-02

**Authors:** Congfeng Li, Ting Ao, Yingxiu Huang, Jinxiang Wang, Lin Ding

**Affiliations:** 1 Department of Pulmonary and Critical Care Medicine, Beijing Luhe Hospital, Capital Medical University, Beijing, China; 2 Department of Infectious Disease, Beijing Luhe Hospital, Capital Medical University, Beijing, China

**Keywords:** Platelet count, Community acquired pneumonia, Dryad database, Glucocorticoids, Mortality, Biomarkers, Diseases, Medical research, Risk factors

## Abstract

Platelet count is a readily available hematological parameter that has been recognized as a potential indicator of inflammation and thrombosis. However, previous research has not examined the prognostic significance of platelet count in patients with community-acquired pneumonia (CAP) undergoing systemic glucocorticoid therapy. This study seeks to explore the correlation between admission platelet count and 30-day mortality rates among CAP patients who are under systemic glucocorticoids treatment. Data were sourced from the Dryad database. The study population included patients diagnosed with CAP who had received systemic glucocorticoids prior to hospitalization. Patients were stratified into tertiles based on their admission platelet count. We utilized multivariable Cox regression models to evaluate the independent effect of platelet count and 30-day mortality, with adjustments for potential confounding variables. Kaplan-Meier survival analysis was conducted to compare survival rates among these groups. Complementing the primary analyses, restricted cubic spline curves and subgroup analyses were implemented to appraise the association between platelet counts and 30-day mortality among diverse subgroups. Additionally, a sensitivity analysis was performed to substantiate the robustness of our findings. A total of 614 CAP patients were included in the study. The 30-day mortality rate was 21.3% (131/614). Platelet count exhibited a significant association with mortality. Multivariable Cox regression analysis indicated that higher platelet count was independently associated with reduced 30-day mortality (HR: 0.94, 95% CI: 0.92–0.97, *P* < 0.001), indicating that each per 10 × 10^9^/L rise in platelet count was associated with a 6% reduce in mortality risk. This association remained significant after adjusting for potential confounders (HR: 0.96, 95% CI: 0.94–0.99, *P* = 0.002). Patients in tertile 2 demonstrated a non-significant trend toward reduced mortality (HR 0.60, 95% CI: 0.35–1.02, *P* = 0.061), whereas those in tertile 3 showed a significantly lower risks of 30-day mortality compared to tertile 1 (HR 0.51, 95% CI: 0.31–0.86, *P* = 0.012). Kaplan-Meier survival curves showed that patients in the highest platelet count tertile had the highest survival rates (log-rank test: *P* < 0.001). Subgroup analyses revealed consistent results across different subgroups. Platelet count was inversely linearly associated with 30-day mortality. Higher platelet count on admission is significantly associated with reduced 30-day mortality in CAP patients receiving systemic glucocorticoids. This finding suggests that platelet count could serve as a potential prognostic marker for early risk stratification and clinical decision-making in this patient population.

**Supplementary Information:**

The online version contains supplementary material available at 10.1038/s41598-026-46565-z.

## Introduction

Community-acquired pneumonia (CAP) is a globally prevalent infectious disease that imposes a significant burden on healthcare systems and leads to high morbidity and mortality rates^1,2^. Patients with underlying conditions such as connective tissue diseases such as systemic lupus erythematosus (SLE), Rheumatoid Arthritis (RA) and Vasculitis, chronic pulmonary diseases, chronic glomerulonephritis or nephrotic syndrome, allergic disease, organ transplantation, along with hematologic disorders often require systemic glucocorticoid therapy^3–8^. Systemic use of high-dose glucocorticoids can lead to severe immunosuppression and increased risk of severe infection and mortality^9,10^. These patients are at a significantly increased risk of developing pulmonary infections, including CAP. Accurate assessment of pneumonia severity and prognosis is crucial for guiding clinical decision-making and optimizing patient outcomes. Hence, there is a necessity for dependable scoring systems or indicators to steer clinical choices and evaluate the prognosis of patients with pneumonia.

Currently, the Pneumonia Severity Index (PSI) and CURB-65 (Confusion, Urea nitrogen, Respiratory rate, Blood pressure, Age ≥ 65 years) score are extensively utilized in clinical settings to evaluate disease severity and prognosis in patients with CAP who have normal immune function. However, the prognostic accuracy of these tools seems to be diminished in immunosuppressed populations^11^, particularly in CAP patients undergoing systemic glucocorticoid therapy. Consequently, it is imperative to identify novel and effective prognostic markers to accurately assess the mortality risk in this population.

Platelet count is an easily obtainable hematological parameter that reflects the body’s inflammatory and thrombotic status. Beyond their role in hemostasis, platelets are immune cells and are actively involved in combating infections^12–14^. Recent studies have shown that platelet count may have prognostic value in various clinical conditions such as chronic obstructive pulmonary disease (COPD), COVID-19, infective endocarditis, stroke, acute respiratory failure and sepsis^15–21^. In the context of CAP, platelet count may provide additional information regarding disease severity and the risk of complications. However, the relationship between platelet count and mortality in CAP patients receiving systemic glucocorticoid therapy has not been extensively studied. Therefore, the aim of this study is to explore the relationship between admission platelet count and 30-day mortality in patients with community-acquired pneumonia (CAP) who are receiving systemic glucocorticoids.

## Methods

### Data source and study population

The study utilized data from the Dryad public database (https://datadryad.org/stash), which offers unrestricted access to the dataset employed in a prior study conducted by Li et al. in 2020^9^. This investigation adopts a multicenter, retrospective, observational design. The dataset included 614 patients diagnosed with CAP who had received systemic glucocorticoids therapy including oral or intravenous glucocorticoids before hospital admission. The study was executed across six academic hospitals, encompassing both secondary and tertiary care levels, located in China. The data collection period spanned from January 2013 to December 2017. Ethical approval for the studies involving human participants was obtained from the Ethics Committee of China-Japan Friendship Hospital. The research was carried out in strict compliance with local legislation and institutional requirements. Furthermore, the execution of the entire study strictly adhered to all relevant guidelines and operational protocols.

Inclusion criteria for our study were as follows: (1) patients aged 18 years or older; (2) diagnosis of CAP based on American Thoracic Society and Infectious Disease Society of America guidelines^22^; (3) receipt of systemic glucocorticoids including oral or intravenous glucocorticoids before hospital admission. Exclusion criteria included: (1) hospital-acquired pneumonia; (2) missing values for platelet count; (3) inability to provide informed consent.

### Variables and definitions

Participants were stratified into tertiles based on admission platelet count. The platelet count tertiles were defined as follows: (1) T1: <149 × 10^9^/L; (2) T2: 149–221 × 10^9^/L; (3) T3: ≥221 × 10^9^/L. Other variables included demographic data (age, gender), medical history (hypertension, coronary heart disease, chronic heart failure, diabetes mellitus, chronic lung disease, including idiopathic interstitial pneumonia, interstitial lung disease, chronic obstructive pulmonary disease, asthma, and bronchiectasis, chronic renal disease or nephrotic syndrome, cerebrovascular diseases and connective tissue disease), severity of illness (PSI score, CURB-65 score), Laboratory data (such as white blood cell count, lymphocyte, hemoglobin, platelet, albumin, lactate dehydrogenase, creatinine, blood urea nitrogen, glucose, Na, erythrocyte sedimentation rate, procalcitonin, prothrombin time, oxygenation index, persistent lymphocytopenia), and treatment-related variables (high-dose glucocorticoid, cumulative methylprednisolone dosages, Intubation, ICU admission, extracorporeal membrane oxygenation(ECMO), continuous veno-venous hemofiltration(CVVH)). Survival status was evaluated at 30 - day post-admission intervals. Meanwhile, all laboratory tests were conducted within 24 h of admission.

Persistent lymphocytopenia was defined as a peripheral blood lymphocyte count of less than 1 × 10⁹/L for more than 7 days. High-dose steroid use was defined as a daily dose of at least 30 mg of prednisolone or its glucocorticoid equivalent within 30 days prior to admission^9^. The glucocorticoid accumulation was defined as the cumulative glucocorticoid exposure. Specifically, it refers to the total dose of prednisone or its equivalent including oral or intravenous glucocorticoids administered to the patients from the start of glucocorticoid therapy until the point of current hospital admission. The data source and detailed extraction methods for all key variables are summarized in Supplementary Table 1.

The primary outcome was 30-day mortality.

### Statistical analysis

Participants were divided into three groups according to the tertiles of the platelet count. Categorical variables were presented as frequencies and percentages. Continuous variables were expressed as mean ± standard deviation (SD) or median (interquartile range, IQR), depending on the distribution. In our analysis of baseline characteristics, continuous variables were compared using the Student’s t-test or the Mann–Whitney U test, as appropriate, while categorical variables were assessed with the chi-square test. For variables with missing data rates below 40%, the K-Nearest Neighbors (KNN) imputation method was employed^23^. For ease of clinical interpretation, the platelet count was transformed using a multiplicative factor (per 10 × 10^9^/L) before analysis. Variables were selected for inclusion in the multivariate Cox proportional hazards models based on clinical relevance, univariate regression results, and a change in the effect estimate exceeding 10%.

The association between platelet count and 30-day mortality was assessed using Kaplan-Meier survival analysis and multivariable Cox regression models. Multivariable cox regression models were adjusted for potential confounders, and the lowest tertile of platelet count served as the reference group. Model I: Unadjusted. Model II: Adjusted for age, gender, coronary heart disease, chronic renal failure or nephrotic syndrome, hypertension, chronic heart failure, diabetes mellitus, chronic lung disease. Model III: Adjusted for age, gender, coronary heart disease, chronic renal failure or nephrotic syndrome, hypertension, chronic heart failure, diabetes mellitus, chronic lung disease, oxygenation index, creatinine, procalcitonin, persistent lymphocytopenia. Model IV: Adjusted for age, gender, nephrotic syndrome or chronic renal failure, coronary heart disease, hypertension, chronic heart failure, diabetes mellitus, chronic lung disease, oxygenation index, creatinine, procalcitonin, persistent lymphocytopenia, Intubation, glucocorticoid accumulation, CVVH. The relationship between platelet count and 30-day mortality was observed and analyzed using restricted cubic splines. Subgroup analyses were performed based on age, gender, diabetes mellitus (Yes or No), CURB 65 (< 2 or ≥ 2), chronic lung disease (Yes or No), and nephrotic syndrome or chronic renal failure (Yes or No). Furthermore, sensitivity analyses were conducted, excluding patients with liver cirrhosis and leukemia.

All statistical tests were two-tailed, with a significance level of *P* < 0.05. Data analysis was performed utilizing R (version 4.2.3) (http://www.R-project.org, The R Foundation) and Free Statistics software (version 2.1, Beijing, China) which is a user-friendly tool built on R language.

## Results

### Baseline characteristics of the study

**Fig. 1:**
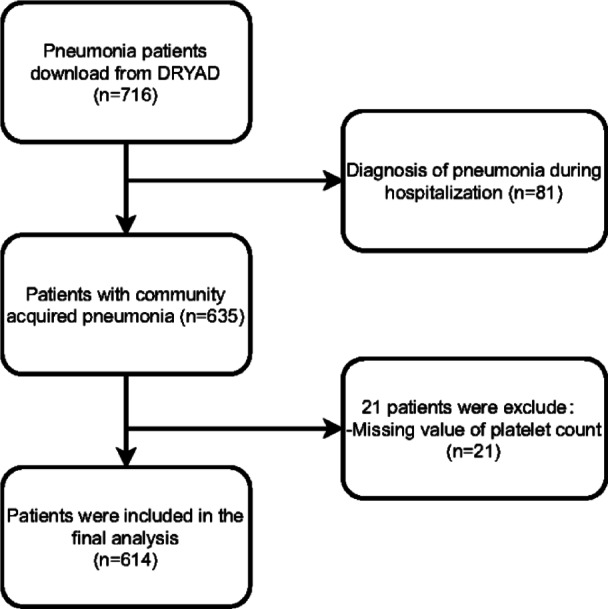
Flowchart of the study.

A total of 614 CAP patients were included in the final study (Fig. 1). Participants aged 60 years or older accounted for 52.8%, and males accounted for 53.6%. The most common underlying comorbidities were chronic lung disease (61.4%) and connective tissue disease (52.6%). The mean platelet count upon admission was (192 ± 89.9) × 10^9^/L. The 30-day mortality rate was 21.3% (131/614). Table 1 details baseline characteristics of all participants.

**Table 1:** Baseline characteristics of the study population based on platelet count tertiles.

Variables	Total (*n* = 614)	T1 (*n* = 205) <149(x10^9^/L)	T2 (*n* = 200) 149–221(x10^9^/L)	T3 (*n* = 209) > 221(x10^9^/L)	*p*
Age, ≥ 60years, n (%)	324 (52.8)	127 (62)	103 (51.5)	94 (45)	0.002
Age, n (%)					
18–39	85 (13.8)	24 (11.7)	27 (13.5)	34 (16.3)	0.066
40–49	79 (12.9)	25 (12.2)	22 (11)	32 (15.3)	
50–59	126 (20.5)	29 (14.1)	48 (24)	49 (23.4)	
60–69	187 (30.5)	78 (38)	58 (29)	51 (24.4)	
70–79	94 (15.3)	31 (15.1)	32 (16)	31 (14.8)	
80–99	43 ( 7.0)	18 (8.8)	13 (6.5)	12 (5.7)	
Gender, Male, n (%)	329 (53.6)	116 (56.6)	119 (59.5)	94 (45)	0.008
Hypertension, n (%)	214 (34.9)	81 (39.5)	73 (36.5)	60 (28.7)	0.059
Coronary heart disease, n (%)	77 (12.5)	33 (16.1)	27 (13.5)	17 (8.1)	0.044
Chronic heart failure, n (%)	16 ( 2.6)	9 (4.4)	3 (1.5)	4 (1.9)	0.014
Diabetes mellitus, n (%)	152 (24.8)	56 (27.3)	50 (25)	46 (22)	0.455
Chronic lung disease, n (%)	377 (61.4)	117 (57.1)	132 (66)	128 (61.2)	0.182
Nephrotic syndrome or CRF, n (%)	100 (16.3)	32 (15.6)	33 (16.5)	35 (16.7)	0.947
Cerebrovascular diseases, n (%)	46 ( 7.5)	17 (8.3)	10 (5)	19 (9.1)	0.253
Connective tissue disease, n (%)	323 (52.6)	114 (55.6)	104 (52)	105 (50.2)	0.538
WBC (x10^9^/L), Median (IQR)	8.0 (5.8, 11.6)	6.7 (4.6, 9.1)	8.1 (6.1, 12.0)	10.1 (7.1, 12.9)	< 0.001
Lymphocyte(x10^9^/L), Median (IQR)	0.9 (0.5, 1.4)	0.7 (0.4, 1.2)	0.8 (0.5, 1.3)	1.1 (0.7, 1.6)	< 0.001
Hemoglobin(g/L), Mean ± SD	113.2 ± 23.6	105.8 ± 26.1	117.3 ± 22.6	116.3 ± 20.1	< 0.001
Platelet(x10^9^/L), Mean ± SD	192.0 ± 89.9	99.7 ± 38.5	184.4 ± 19.7	289.8 ± 62.7	< 0.001
Albumin(g/L), Mean ± SD	32.8 ± 6.2	31.6 ± 6.7	33.9 ± 5.7	33.0 ± 6.0	0.002
LDH (U/L), Median (IQR)	313.0 (225.0, 476.5)	318.0 (233.2, 520.8)	334.0 (231.5, 476.5)	297.0 (216.0, 416.5)	0.128
BUN(mmol/L), Median (IQR)	6.2 (4.6, 9.6)	7.5 (5.0, 13.1)	6.0 (4.7, 9.3)	5.6 (4.1, 7.9)	< 0.001
Creatinine(mmol/L), Median (IQR)	65.0 (51.8, 90.7)	69.3 (52.8, 121.7)	64.8 (52.9, 88.2)	63.6 (50.2, 80.2)	0.014
Glucose (mmol/L), Median (IQR)	5.7 (4.6, 8.0)	6.4 (4.8, 8.8)	5.7 (4.5, 8.1)	5.1 (4.4, 7.0)	0.001
NA, Mean ± SD	137.5 ± 6.6	137.0 ± 5.9	137.3 ± 4.8	138.1 ± 8.4	0.241
ESR (mm/h), Median (IQR)	39.0 (19.0, 68.0)	34.0 (16.0, 69.0)	38.0 (19.0, 65.5)	48.0 (27.0, 73.0)	0.111
Procalcitonin (ng/mL), Median (IQR)	0.3 (0.1, 0.7)	0.3 (0.1, 1.1)	0.2 (0.1, 0.7)	0.3 (0.1, 0.5)	0.019
Prothrombin time(sec), Median (IQR)	13.8 (12.7, 16.9)	14.0 (12.4, 17.4)	13.4 (12.9, 15.4)	13.8 (12.7, 19.1)	0.669
Oxygenation index, Median (IQR)	248.0 (136.2, 351.1)	229.9 (114.8, 342.0)	250.0 (127.6, 371.4)	259.0 (152.9, 349.2)	0.123
Persistent lymphocytopenia, n (%)	259 (42.2)	112 (54.6)	81 (40.5)	66 (31.6)	< 0.001
Glucocorticoid accumulation, Median (IQR)	4.0 (2.2, 8.8)	3.6 (2.0, 9.0)	3.9 (2.2, 8.1)	4.3 (2.2, 10.0)	0.667
High dose glucocorticoid, n (%)	216 (35.2)	76 (37.1)	65 (32.5)	75 (35.9)	0.607
CURB65 > 1, n (%)	176 (28.7)	84 (41)	54 (27)	38 (18.2)	< 0.001
PSI, Mean ± SD	80.3 ± 31.1	87.2 ± 31.7	79.0 ± 30.4	74.8 ± 29.9	< 0.001
Intubation, n (%)	127 (24.7)	52 (30.4)	39 (22.8)	36 (20.8)	0.094
ICU admmission, n (%)	247 (40.2)	97 (47.3)	77 (38.5)	73 (34.9)	0.031
ECMO, n (%)	29 ( 4.7)	9 (4.4)	10 (5)	10 (4.8)	0.958
Vasoactive drugs, n (%)	102 (16.6)	44 (21.6)	31 (15.5)	27 (12.9)	0.054
CVVH, n (%)	58 ( 9.4)	30 (14.6)	13 (6.5)	15 (7.2)	0.008
30-Day mortality, n (%)	131 (21.3)	63 (30.7)	39 (19.5)	29 (13.9)	< 0.001

### Kaplan-Meier survival analysis

**Fig. 2:**
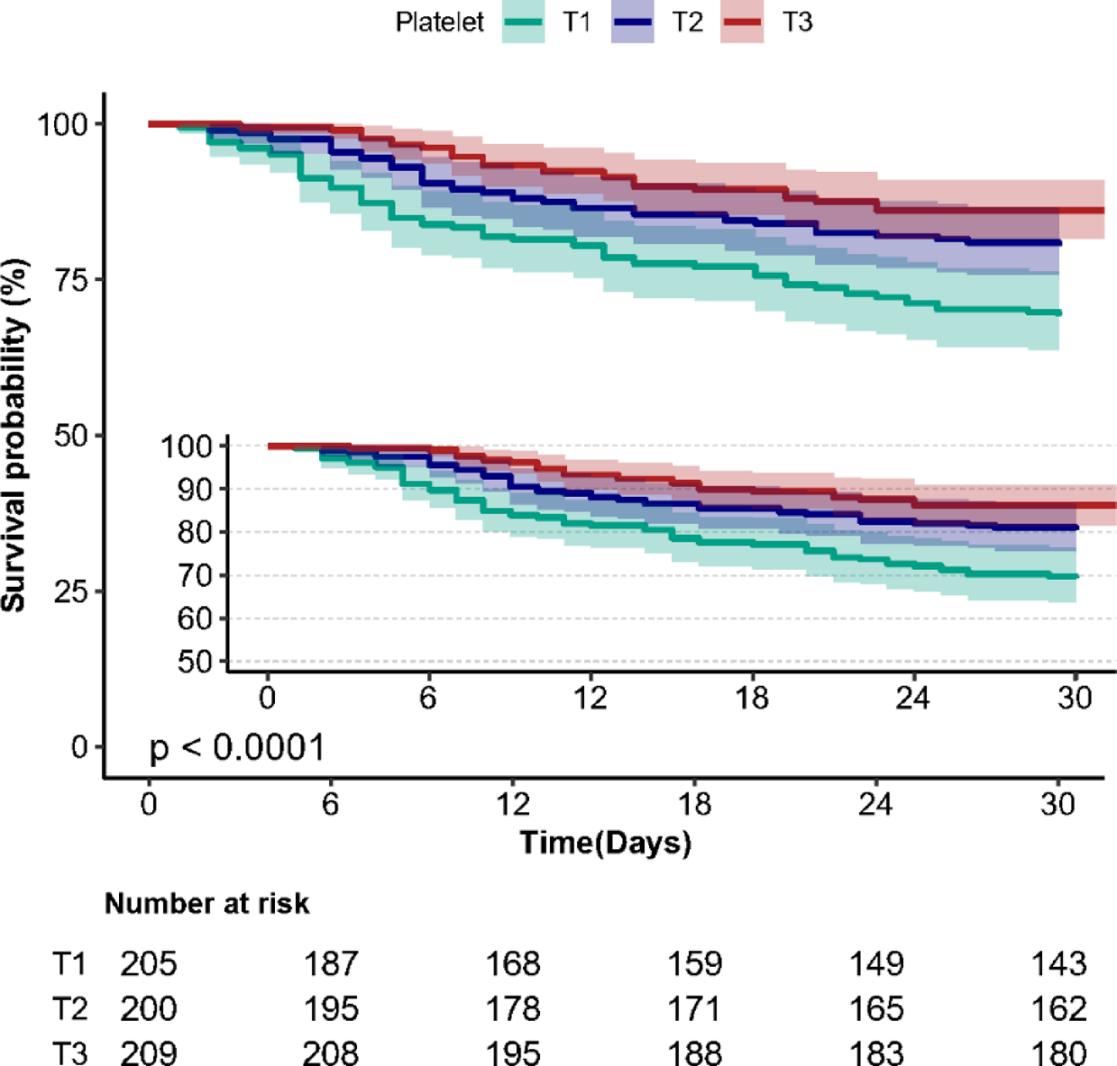
Kaplan-Meier survival curves for day 30 of patients depending on the tertile of platelet.

Kaplan–Meier survival curves revealed a significant difference in 30-day survival rates among patients across different platelet count tertiles (log-rank test: *P* < 0.001). Specifically, patients in the highest platelet count tertile (T3) exhibited the highest survival rates, compared to those in the lowest tertile (T1) (Fig. 2).

### Multivariable cox regression analysis

Multivariable Cox regression analysis indicated that higher platelet count was independently associated with reduced 30-day mortality (HR: 0.94, 95% CI: 0.92–0.97, *P* < 0.001), indicating that each per 10 × 10^9^/L rise in platelet count was linked to a 6% reduce in mortality risk. This association remained significant after adjusting for potential confounders (HR 0.96, 95% CI: 0.94–0.99, *P* = 0.003). Patients in tertile 2 demonstrated a non-significant trend toward reduced mortality (HR 0.60, 95% CI: 0.35–1.02, *P* = 0.061), whereas those in tertile 3 showed a significantly lower risks of 30-day mortality compared to tertile 1 (HR 0.51, 95% CI: 0.31–0.86, *P* = 0.012) (Table 2).

**Table 2:** Multivariable Cox regression to assess the association of platelet count with 30-day mortality.

Variable	Model I		Model II		Model III		Model IV		
	HR (95% CI)	*P*-value	HR (95% CI)	*P*-value	HR (95% CI)	*P*-value		*P*-value	
Platelet (per 10 × 10^9^/L)		0.94 (0.92 ~ 0.97)	< 0.001	0.94 (0.92 ~ 0.97)	< 0.001	0.96 (0.94 ~ 0.98)	< 0.001	0.96 (0.94 ~ 0.99)	0.003
Platelet tertiles (per 10 × 10^9^/L)									
T1	1(Ref)		1(Ref)		1(Ref)		1(Ref)		
T2	0.59 (0.39 ~ 0.87)	0.009	0.57 (0.38 ~ 0.85)	0.007	0.66 (0.42 ~ 1.02)	0.063	0.60 (0.35 ~ 1.02)	0.061	
T3	0.4 (0.26 ~ 0.62)	< 0.001	0.4 (0.25 ~ 0.63)	< 0.001	0.48 (0.3 ~ 0.77)	0.002	0.51 (0.31 ~ 0.86)	0.012	
P for trend		< 0.001		< 0.001		0.002		0.012	

### Restricted cubic spline analysis

Additionally, after adjusting for confounders using Cox regression in Model IV, restricted cubic spline analysis validated a linear relationship between platelet count and 30-day mortality, with a non-linearity *P*-value of 0.696. The mortality risk showed a decreasing trend in conjunction with increasing platelet count, as illustrated in Supplementary File 1.

### Subgroup analyses

**Fig. 3:**
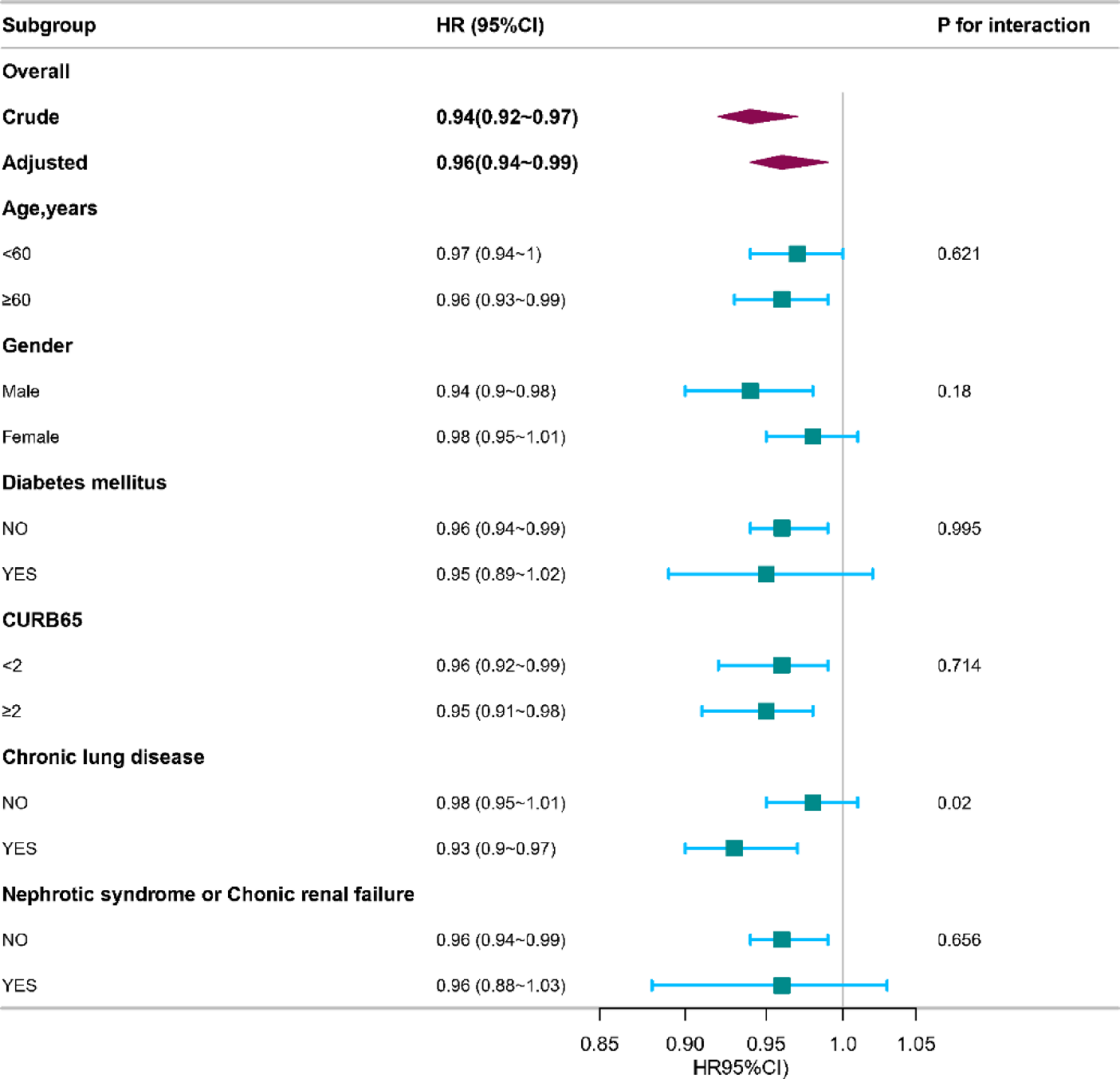
Subgroup analysis of platelet count and 30-day mortality in community-acquired pneumonia. Adjusted for age, gender, coronary heart disease, chronic renal failure or nephrotic syndrome, hypertension, chronic heart failure, diabetes mellitus, chronic lung disease, oxygenation index, creatinine, procalcitonin, persistent lymphocytopenia, Intubation, glucocorticoid accumulation, CVVH.

Subgroup analyses revealed consistent results across different subgroups, including age, gender, diabetes mellitus, CURB 65, chronic lung disease, and chronic renal failure or nephrotic syndrome (Fig. 3). Interaction analysis indicated that chronic lung disease significantly interacted with platelet count in predicting 30-day mortality (*P* < 0.05). Interaction analysis revealed that chronic lung disease significantly modified the association between platelet count and 30-day mortality (*P* for interaction = 0.02). Specifically, in the subgroup of patients with chronic lung disease, a higher platelet count per 10 × 10⁹/L increment was associated with a 7% reduction in mortality risk (HR 0.93, 95% CI: 0.90–0.97). Conversely, in patients without chronic lung disease, the association was attenuated and not statistically significant (HR 0.98, 95% CI: 0.95–1.01).

### Sensitivity analysis

To verify the robustness of our results, we conducted a sensitivity analysis. After excluding 13 patients with leukemia or cirrhosis, sensitivity analysis confirmed that platelet count significantly correlates with 30-day mortality in CAP patients undergoing systemic glucocorticoid therapy (Table 3).

**Table 3:** Multivariable Cox regression to assess the association of platelet count with 30-day mortality (Exclude 13 patients with leukemia and cirrhosis, final 601 patients included**).**

Variable	Model I		Model II	
	HR(95% CI)	*P*-value		*P*-value
Platelet (per 10 × 10^9^/L)	0.95 (0.93 ~ 0.97)	< 0.001	0.96 (0.94 ~ 0.99)	0.002
Platelet tertiles (per 10 × 10^9^/L)				
T1(< 15)	1(Ref)		1(Ref)	
T2(15−22.2)	0.62 (0.41 ~ 0.92)	0.018	0.56 (0.32 ~ 0.96)	0.035
T3(> 22.2)	0.41 (0.26 ~ 0.64)	< 0.001	0.45 (0.26 ~ 0.77)	0.004
P for trend		< 0.001		0.004

## Discussion

This retrospective analysis reveals a significant correlation between elevated platelet count at admission and decreased mortality rates within 30 days in CAP patients treated with systemic glucocorticoids. This relationship is visually represented by the restricted cubic spline curve. Kaplan–Meier survival analysis further supports this, indicating that higher platelet count is significantly associated with a reduced risk of 30-day mortality compared to those with lower counts. Subgroup analyses across various patient subgroups consistently reinforce these findings. These results suggest that platelet count may serve as a valuable prognostic indicator for early risk assessment in CAP patients undergoing systemic glucocorticoid therapy.

Platelets are anucleate cell fragments derived from megakaryocytes and play a key role in multiple pathophysiological mechanisms, including hemostasis, thrombosis, inflammation, and antimicrobial defense^24^. Previous studies have investigated the role of platelet count in various clinical contexts, including infections, inflammatory and malignant diseases, highlighting its potential as a prognostic indicator. In one large, nested case–control study, an elevated platelet count was found to be associated with an increased risk of developing new solid tumors^25^. Recent research has established that a diminished peak platelet count serves as a robust predictor of 50-day mortality^26^. Additionally, it has been reported that both thrombocytopenia and thrombocytosis are associated with mortality in CAP patients^27^. Guillaume M et al. further revealed that platelet counts, even within the normal range, are associated with mortality in adult patients hospitalized for CAP^28^. In a study by Gorelik O, it was discovered that among patients hospitalized with CAP, an increasing platelet count is associated with favorable outcomes, whereas a decreasing platelet count is linked to a poor prognosis. Therefore, monitoring changes in platelet count can provide useful prognostic insights^29^. Chen Y et al. found that lower platelet counts in H7N9 patients are associated with higher mortality risks^30^. In a separate study, Yang Q revealed a U-shaped association between platelet count and in-hospital mortality in patients with COVID-19, indicating that both low and high platelet counts are linked to increased mortality risks^16^. Previous other studies have also suggested a nonlinear relationship between platelet count and the prognosis of various diseases^15,17,19,31^. One previous study has also shown that thrombocytosis in CAP patients is associated with adverse outcomes, complicated pleural effusion, and empyema^32^. In contrast to the study by Prina et al., which identified thrombocytosis as a marker of poor outcome in a general CAP population, our study revealed a protective association between higher platelet count and reduced 30-day mortality. Specifically, we observed a distinct linear relationship rather than a nonlinear one in this specific cohort of patients on systematic glucocorticoid therapy. This discrepancy may be attributed to the differences in study populations. Our study identifies a significant inverse linear association between platelet count and 30-day mortality in CAP patients receiving systemic glucocorticoids, which contrasts with the U-shaped or nonlinear relationships reported in studies of immunocompetent general CAP populations. This discrepancy can be mechanistically explained by the unique context of glucocorticoid-induced immunosuppression, which may reshape the prognostic significance of platelet counts through several distinct pathways. First, regarding platelet production, recent evidence indicates that glucocorticoids stimulate thrombopoiesis by remodeling the megakaryocyte transcriptome and promoting proplatelet formation^33^. Therefore, in our immunosuppressed cohort, a higher platelet count may not reflect a pathological state but rather a preserved bone marrow reserve and a compensatory response to infection, indicating a less severe state of immune exhaustion. Second, concerning platelet function, glucocorticoids exert a suppressive effect on excessive platelet activation and pro-inflammatory responses. Specifically, they inhibit thromboxane A2 generation by regulating cPLA2 phosphorylation, thereby dampening the positive feedback of platelet aggragation^34^. This suppression of systemic hyperinflammation may paradoxically preserve the functional integrity of platelets, allowing them to maintain their beneficial role in immunothrombosis. In the context of CAP, this could enable effective pathogen containment within the pulmonary vasculature without precipitating widespread deleterious thrombosis or the dysregulated inflammation often seen in immunocompetent hosts. Third, platelets play a vital role in maintaining endothelial integrity and promoting repair after vascular injury. In our immunocompromised patients, the anti-inflammatory effects of glucocorticoids may paradoxically create a context where platelet-mediated endothelial support becomes disproportionately important. We hypothesize that a preserved platelet count may help offset the potential endothelial dysfunction associated with systematic glucocorticoid use and the direct assault from pulmonary infection, thereby contributing to the observed survival benefit.

The contrasting finding from Prina et al., where thrombocytosis predicted poor outcomes in a general CAP cohort, underscores the critical effect of population characteristics and helps define the applicability boundary of our results. In immunocompetent hosts, high platelet counts are often driven by underlying chronic inflammation or malignancies, which are inherently associated with high mortality and constitute the right arm of the U-shaped curve. Conversely, in our cohort of patients with drug-induced immunosuppression, a higher platelet count is more likely to signify a retained capacity to mount an appropriate hematopoietic and immunothrombotic response to infection. Therefore, the prognostic meaning of platelet count is fundamentally reshaped by the patient’s immune status. Our findings suggest that the protective linear association is specific to CAP patients with glucocorticoid-induced immunosuppression and may not extend to immunocompetent individuals.

Use of systemic glucocorticoids can lead to immunosuppression and increased risk of treatment-resistant pneumonia and mortality. Moreover, glucocorticoids may affect platelet function and count. One study has shown that glucocorticoids can alter platelet activation and aggregation, which in turn affects their role in inflammation and coagulation^33^. Platelets play a crucial role in the immune response by releasing cytokines and chemokines that enhance the recruitment and activation of immune cells^35^. In the context of CAP, a higher platelet count may indicate a more effective immune response, leading to better control of the infection and reduced mortality. Additionally, platelets contribute to vascular integrity and repair^36^, which may be particularly important in patients with underlying chronic conditions and immune suppression due to systmic glucocorticoid use. This may explain why the association between platelet count and mortality is more pronounced in CAP patients receiving systemic glucocorticoids. The specific mechanisms may require further investigation. Moreover, the sample size of the study is relatively small, so conclusions should be drawn with caution.

A key finding is the significant effect modification by chronic lung disease status, wherein the protective association of a higher platelet count was more pronounced in patients with pre-existing chronic lung disease. We hypothesize that this subgroup, characterized by impaired mucociliary clearance and compromised local immunity may rely more heavily on platelet-dependent host defense mechanisms, such as direct antimicrobial activity and immunothrombosis, to contain pulmonary infection. Consequently, a preserved platelet count may serve as a crucial marker of intact reserve capacity in this vulnerable population, explaining the stronger survial benefit. Conversely, in patients without chronic lung disease, redundant and intact respiratory defenses may diminish the relative prognostic weight of platelet count alone.

This study offers several notable strengths. Firstly, it leverages a cohort from the Dryad database, which, despite not being exceedingly large, provides a focused and detailed dataset with rich clinical information regarding the CAP patients receiving systemic glucocorticoids. Secondly, the study employs straightforward and easily obtainable indicators, such as platelet count, which are readily accessible in routine clinical settings. This simplicity not only facilitates widespread application but also enhances the potential for adoption in primary care settings where resources may be limited. Thirdly, the comprehensive analytical approach, including multivariable Cox regression models and Kaplan-Meier survival analysis, ensures a thorough evaluation of the relationship between platelet count and mortality. Finally, subgroup analyses and sensitivity tests further solidify the robustness and reliability of the findings. However, this study also has several limitations. Firstly, the sample size is relatively small, which may limit the generalizability of the findings and the statistical power to detect significant associations. Secondly, the retrospective design may introduce information biases, as we were unable to precisely ascertain the exact initiation time and cumulative dosage of glucocorticoid therapy for each patient, which could affect the accuracy and robustness of our findings. Thirdly, the platelet count was measured only at admission and not dynamically assessed over the course of the illness, which may limit the understanding of its temporal relationship with mortality. Future studies should address these limitations by employing larger sample sizes, prospective designs, and more comprehensive data collection to further validate the prognostic value of platelet count in CAP patients receiving systemic glucocorticoids. Dynamic assessment of platelet count over the course of the illness could provide additional insights into its prognostic utility.

## Conclusion

Higher platelet count on admission is significantly associated with reduced 30-day mortality in CAP patients receiving systemic glucocorticoids. This finding suggests that platelet count could serve as a potential prognostic marker for early risk stratification and clinical decision-making in this patient population. Future studies should further investigate the underlying mechanisms and validate the prognostic value of platelet count in larger cohorts.

## Supplementary Information

Below is the link to the electronic supplementary material.

Supplementary Material 1

Supplementary Material 2

## Data Availability

The datasets presented in this study can be found in online repositories (https://datadryad.org/dataset/doi:10.5061/dryad.mkkwh70x2).
