# Supplementary material for: Association between platelet count and 30-day mortality in community-acquired pneumonia patients receiving systemic glucocorticoids therapy
Source: Sci Rep. 2026 Apr 2;16:15605. doi: 10.1038/s41598-026-46565-z (PMC13186940; doi:10.1038/s41598-026-46565-z)
Supplement: Supplementary file 1 — Supplementary Material 1 [file 41598_2026_46565_MOESM1_ESM.docx]

**Supplementary File 1 Association of platelet count with 30-day mortality**


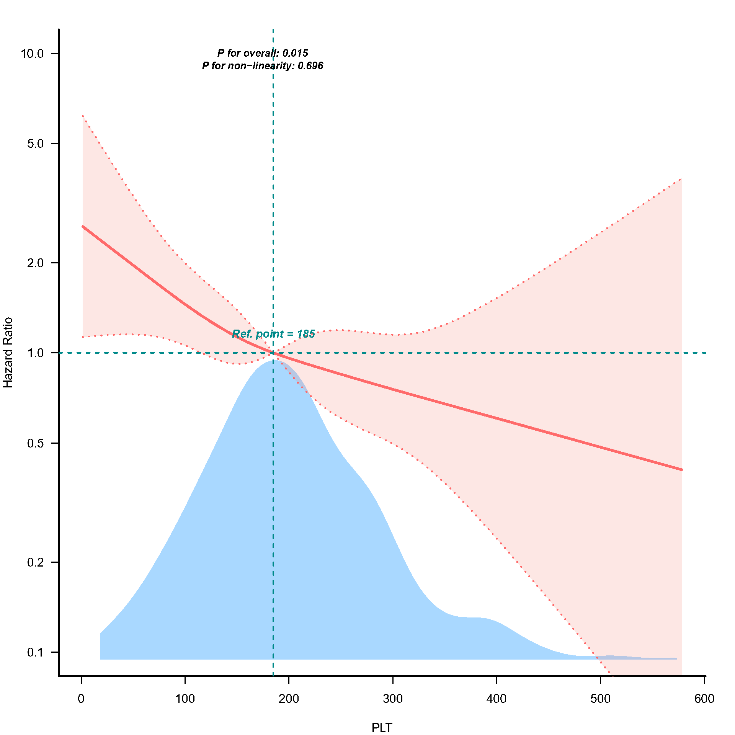


**Figure 1S** Association of platelet count with 30-day mortality. Adjusted for age, gender，coronary heart disease, chronic renal failure or nephrotic syndrome, hypertension, chronic heart failure, diabetes mellitus, chronic lung disease，oxygenation index, creatinine, procalcitonin, persistent lymphocytopenia, Intubation, glucocorticoid accumulation, CVVH. 99% of the data are shown in the figure. Abbreviations: CVVH, continuous veno-venous hemofiltration.
