# Supplementary material for: Association between platelet count and 30-day mortality in community-acquired pneumonia patients receiving systemic glucocorticoids therapy
Source: Sci Rep. 2026 Apr 2;16:15605. doi: 10.1038/s41598-026-46565-z (PMC13186940; doi:10.1038/s41598-026-46565-z)
Supplement: Supplementary file 2 — Supplementary Material 2 [file 41598_2026_46565_MOESM2_ESM.docx]

**Table 1S: Data Source and Extraction Methods for Key Variables**

| **Variable Category** | **Variable Name** | **Data Source / Extraction Method** | **Notes / Specifics** |
| --- | --- | --- | --- |
| Demographics | Age | EHR Structured Fields | Extracted directly from structured patient registration data |
|  | Gender | EHR Structured Fields | Extracted directly from structured patient registration data |
| Medical history | Hypertension | Electronic Health Record | ICD codes, YES=1, NO=0 |
|  | Coronary heart disease | Electronic Health Record | ICD codes, YES=1, NO=0 |
|  | Chronic heart failure | Electronic Health Record | ICD codes, YES=1, NO=0 |
|  | Diabetes mellitus | Electronic Health Record | ICD codes, YES=1, NO=0 |
|  | Chronic lung disease | Electronic Health Record | ICD codes, YES=1, NO=0 |
|  | Chronic renal disease or nephrotic syndrome | Electronic Health Record | ICD codes, YES=1, NO=0 |
|  | Cerebrovascular disease | Electronic Health Record | ICD codes, YES=1, NO=0 |
|  | Connective tissue disease | Electronic Health Record | ICD codes, YES=1, NO=0 |
| Score system | CURB 65 | Electronic Health Record | Total score was calculated from its five individual components (Confusion, Urea, Respiratory rate, Blood pressure, Age ≥65) during the data cleaning stage. Each component was extracted by reviewing vital signs, laboratory results, and clinical notes from the admission period. |
|  | PSI | Electronic Health Record | Total score was calculated from its twenty individual components during the data cleaning stage. Each component was extracted by reviewing vital signs, laboratory results, imaging reports, and clinical notes from the admission period. |
| Laboratory tests | White blood cell | EHR Structured Fields | First available value within 24 hours of admission |
|  | Lymphocyte | EHR Structured Fields | First available value within 24 hours of admission |
|  | Hemoglobin | EHR Structured Fields | First available value within 24 hours of admission |
|  | Platelet | EHR Structured Fields | First available value within 24 hours of admission |
|  | Albumin | EHR Structured Fields | First available value within 24 hours of admission |
|  | Lactate dehydrogenase | EHR Structured Fields | First available value within 24 hours of admission |
|  | Creatinine | EHR Structured Fields | First available value within 24 hours of admission |
|  | Blood urea nitrogen | EHR Structured Fields | First available value within 24 hours of admission |
|  | Glucose | EHR Structured Fields | First available value within 24 hours of admission |
|  | Na | EHR Structured Fields | First available value within 24 hours of admission |
|  | Erythrocyte sedimentation rate | EHR Structured Fields | First available value within 24 hours of admission |
|  | Procalcitonin | EHR Structured Fields | First available value within 24 hours of admission |
|  | Prothrombin time | EHR Structured Fields | First available value within 24 hours of admission |
|  | Oxygenation index | EHR Structured Fields | First available value within 24 hours of admission |
|  | Persistent lymphocytopenia | EHR Structured Fields | Peripheral blood lymphocyte count of less than 1×10⁹/L for more than 7 days |
| Treat-related variables | High-dose glucocorticoid use | EHR Structured Fields | High-dose steroid use was defined as a daily dose of at least 30 mg of prednisolone or its glucocorticoid equivalent within 30 days prior to admission, YES OR NOT, YES=1, NO=0 |
|  | Cumulative methylprednisolone dosages | EHR Structured Fields and medical history | The glucocorticoid accumulation was defined as the cumulative glucocorticoid exposure. Specifically, it refers to the total dose of prednisone equivalent administered to the patient within 30 days prior to the current hospital admission. |
|  | ECMO | EHR Structured Fields | USE OR NOT, USE=1, NOT=0 |
|  | CVVH | EHR Structured Fields | USE OR NOT, USE=1, NOT=0 |
| Survival status | d30 | EHR Structured Fields | 30-day mortality after admission |
|  | day30time | EHR Structured Fields | Time to mortality within 30 days |

**Abbreviations:** EHR, Electronic Medical Record; CURB 65, Confusion, Urea, Respiratory rate, Blood pressure, Age ≥65; PSI, pneumonia severity index; ECMO, Extracorporeal Membrane Oxygenation; CVVH, continuous veno-venous hemofiltration.
